# Key regulators of sensitivity to immunomodulatory drugs in cancer treatment

**DOI:** 10.1186/s40364-021-00297-6

**Published:** 2021-06-05

**Authors:** Shichao Wang, Zhiyue Li, Shaobing Gao

**Affiliations:** 1 The Fifth Affiliated Hospital of Zhengzhou University, No. 3 Kangfu Front Street, 450052 Zhengzhou, China; 2 The Affiliated Cancer Hospital of Zhengzhou University, Henan Cancer Hospital, 127 Dongming Road, Zhengzhou, 450008 China

**Keywords:** Immunomodulatory drugs, CRISPR-Cas9 screening, CRL4^CRBN^ E3 ligase, PROTACs, Multiple myeloma, Ubiquitination, CC-90009

## Abstract

Immunomodulatory drugs (IMiDs) include thalidomide, lenalidomide, and pomalidomide, which have shown significant efficacy in the treatment of multiple myeloma (MM), myelodysplastic syndrome (MDS) with deletion of chromosome 5q (del(5q)) and other hematological malignancies. IMiDs hijack the CRL4^CRBN^ ubiquitin ligase to target cellular proteins for ubiquitination and degradation, which is responsible for their clinical activity in MM and MDS with del(5q). However, intrinsic and acquired resistance frequently limit the efficacy of IMiDs. Recently, many efforts have been made to explore key regulators of IMiD sensitivity, resulting in great advances in the understanding of the regulatory networks related to this class of drugs. In this review, we describe the mechanism of IMiDs in cancer treatment and summarize the key regulators of IMiD sensitivity. Furthermore, we introduce genome-wide CRISPR-Cas9 screenings, through which the regulatory networks of IMiD sensitivity could be identified.

## Background

**Fig. 1:**
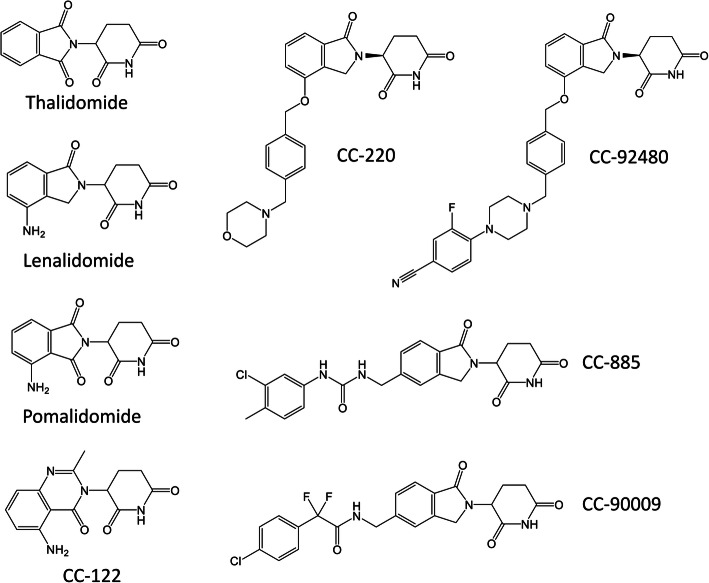
Chemical structures of thalidomide, lenalidomide, pomalidomide, CC-122, CC-220, CC-885, CC-92480 and CC-90009

Thalidomide and its derivatives lenalidomide and pomalidomide are often called immunomodulatory drugs (IMiDs) due to their modulatory effects on immune cells [1–4]. IMiDs have shown remarkable therapeutic efficacy in several hematological malignancies. In combination with steroids, proteasome inhibitors and monoclonal antibodies, IMiDs are widely used to treat multiple myeloma (MM) [5–9]. Lenalidomide also has therapeutic activity in myelodysplastic syndrome (MDS) with deletion of chromosome 5q (del(5q)) [10, 11], mantle cell lymphoma (MCL) [12–15] and chronic lymphocytic leukemia (CLL) [16–18]. New generations of IMiDs, including CC-122 (avadomide) [19–22], CC-220 (iberdomide) [23, 24], CC-885 [25, 26], CC-92480 [27] and CC-90009 [28] (Fig. 1), are being evaluated for their potential to treat diffuse large B-cell lymphoma (DLBCL), follicular lymphoma (FL), MM and acute myeloid leukemia (AML) [29, 30].

Although IMiDs have shown significant efficacy in a range of hematological malignancies, primary and acquired drug resistance limit their clinical application. Thus, it is necessary to delineate the regulatory networks related to IMiD sensitivity. Recently, emerging evidence has shown that sensitivity to IMiDs is regulated by several factors, including CRBN, the Cullin-RING ligase 4 (CRL4) E3 ubiquitin ligase [31–34], RUNX proteins [35], and Wnt/β-Catenin pathway members [36]. Moreover, genome-scale CRISPR screenings have identified a series of key regulators of sensitivity to IMiDs [37–41].

In this review, we highlight the underlying mechanisms of IMiDs in cancer treatment and summarize the key regulators of IMiD sensitivity. Furthermore, we introduce genome-wide CRISPR screenings as a tool that can identify regulatory networks of IMiD sensitivity.

## Mechanism of IMiD activity

**Fig. 2:**
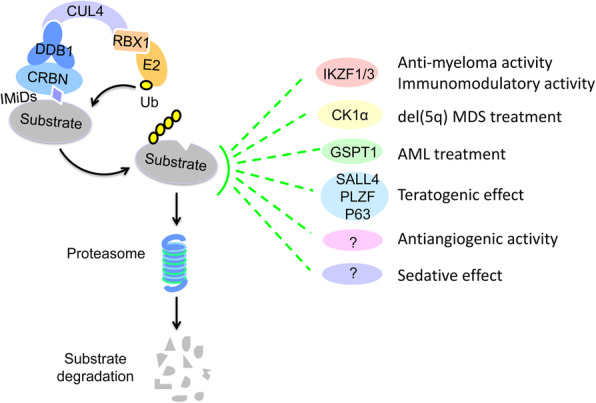
Molecular mechanism of IMiD activity. IMiDs bind CRL4^CRBN^ to recruit multiple neosubstrates for ubiquitination and proteasomal degradation, resulting in pleiotropic effects. CUL4, Cullin 4; Ub, ubiquitin

CRBN, the primary cellular target of IMiDs [42], is a substrate receptor of CRL4, an E3 ubiquitin ligase complex consisting of Cullin 4 A/4B, DNA damage-binding protein 1 (DDB1) and a small RING protein (RBX) [43]. IMiDs hijack the CRL4^CRBN^ E3 ligase to ubiquitinate and degrade two essential lymphoid transcription factors, IKZF1 (Ikaros) and IKZF3 (Aiolos), which leads to the downregulation of IRF4 and MYC, resulting in the toxicity of MM cells [44, 45]. Lenalidomide can bind the CRL4^CRBN^ E3 ligase to induce ubiquitination and degradation of CK1α, accounting for its efficacy in del(5q) MDS [46]. A number of other neosubstrates of IMiDs have been identified using proteomics analysis [47, 48]. Furthermore, the degradation of SALL4, PLZF and P63 proteins was reported to be correlated with thalidomide-induced malformations [48–51]. Thus, this class of compounds are also called CRBN E3 ligase modulators (CELMoDs). IMiDs represent the first class of drugs that function by inducing the degradation of cellular proteins (Fig. 2), which has important implications for the design of novel therapeutic compounds.

## Overview of emerging regulators of sensitivity to immunomodulatory drugs

Since IMiDs bind the CRL4^CRBN^ E3 ligase to ubiquitinate and degrade disease-related proteins, the components of the CRL4^CRBN^ E3 ligase and its activity are hypothesized to be essential for the antitumor activity of IMiDs. Recently, emerging evidence has shown the significance of CRL4^CRBN^ components for IMiD sensitivity, together with other cellular molecules and pathways.

### CRBN

As the primary target of IMiDs, CRBN was reported to be essential for the activity of IMiDs. CRBN knockdown leads to resistance to lenalidomide and pomalidomide in MM cell lines [31]. In addition, lenalidomide- or pomalidomide-resistant MM cells generated by incubation with gradually increasing concentrations of lenalidomide/pomalidomide show a significant decrease in CRBN protein levels [31, 52], suggesting an important role of CRBN in acquired IMiD resistance.

According to several clinical observations, high expression of CRBN has been reported to correlate with improved clinical response to IMiDs in MM patients [32, 33]. In addition, targeted sequencing data have shown that MM patients resistant to IMiDs frequently harbor *CRBN* mutations [53]. *CRBN* alterations, including point mutations, copy loss/structural variations and an exon 10 splice variant transcript, have been found in lenalidomide- or pomalidomide-resistant MM patients [54]. Moreover, approximately one-third of MM patients who are refractory to pomalidomide are reported to carry genetic alterations in *CRBN* [54].

High expression of CRBN is associated with increased clinical efficacy of lenalidomide in del(5q) MDS, while a decrease in CRBN expression correlates with loss of response and disease progression [55]. CRBN expression can also predict clinical response in CLL patients treated with IMiD-based therapy [56].

The above evidence indicates that CRBN expression is required for the antitumor activity of IMiDs. However, a lack of *CRBN* mutations or downregulation of CRBN expression levels has been reported in three MM cell lines intrinsically resistant to IMiDs [57]. In addition, a study reported that only one out of five MM patients refractory to lenalidomide showed significantly low expression of CRBN before treatment [58], indicating that factors other than CRBN might regulate intrinsic resistance to IMiDs.

### CRL4 and IKZF1/3

As IMiDs function through hijacking CRL4^CRBN^ E3 ligase to target neosubstrates like IKZF1/3 for ubiquitination and degradation, the expression of these components is supposed to be a necessity. Cullin 4 proteins consist of two homogenous members, Cullin 4 A and Cullin 4B, which serve as scaffolds for the CRL4 E3 ligase [43]. Mounting evidence has shown that Cullin 4 A and Cullin 4B proteins can promote tumorigenesis in a number of malignancies [59–62]. Overexpression of Cullin 4 A in thalidomide-resistant prostate cancer cells can restore sensitivity to thalidomide, while knockdown of this gene in thalidomide-sensitive 22RV1 cells leads to drug resistance [34]. In addition, mutations in *Cullin 4B* have been found in MM cases with acquired IMiD resistance, as have mutations in *CRBN*, *DDB1* and *IKZF1/3* [63].

IKZF1 (Q146H) and IKZF3 (Q147H) mutants are resistant to lenalidomide-induced degradation, and overexpression of either mutant protein can cause resistance to lenalidomide in MM1S cells [44, 45]. IKZF1 expression is decreased in IMiD-resistant MM cell lines, while MM patients with low expression of IKZF1 show a lack of response to IMiD treatment with shorter overall survival than patients with high expression of IKZF1 [64, 65]. IKZF3 expression predicts favorable response to lenalidomide and high expression of IKZF1/3 is correlated with longer median progression free survival in MM [66]. Moreover, alterations in *IKZF3* at diagnosis have been reported, suggesting that *IKZF3* mutations may contribute to the pathogenesis of MM [63].

### RUNX proteins

The RUNX family of transcription factors, composed of RUNX1, RUNX2 and RUNX3, are highly conserved and form heterodimers with CBFβ to regulate target gene expression during development and hematopoiesis [67–70]. Aberrations in *RUNX* have been frequently identified in leukemia and solid tumors [71–74]. Recently, RUNX proteins have been found to interact and protect IKZF1 and IKZF3 proteins from lenalidomide-induced ubiquitination and degradation, resulting in the desensitization of MM cells to lenalidomide. Inhibition of RUNX proteins by the small molecule AI-10-104 leads to sensitization to lenalidomide in MM cell lines and primary MM cells [35], providing a reference for the combined use of RUNX inhibitors and IMiDs in MM treatment.

In contrast, loss of function of *RUNX1* causes lenalidomide resistance in del(5q) MDS cells, suggesting that RUNX1 function is required for lenalidomide sensitivity [75, 76]. Recurrent variants of *RUNX1* have been discovered in del(5q) MDS patients who become resistant to lenalidomide. Furthermore, RUNX1 forms a complex with GATA2 to drive megakaryocytic differentiation, which is required for lenalidomide efficacy [75]. Thus, RUNX proteins seem to have contrasting impacts on lenalidomide sensitivity in MM and del(5q) MDS cells.

### MEK/ERK

Ras/RAF/MEK/ERK (mitogen-activated protein kinase, MAPK) signaling regulates cellular proliferation, differentiation and survival. Aberrant activation of the MAPK pathway is frequently observed in human cancers, and small molecules targeting this pathway have been approved to treat cancers, including melanoma, colorectal cancer and non-small-cell lung cancer [77, 78]. In a xenograft MM mouse model, acquired resistance to lenalidomide and pomalidomide is developed by continuous administration of pomalidomide-dexamethasone (PD), lenalidomide-dexamethasone (LD) or vehicle [79]. Upregulation of the MEK/ERK pathway has been found in IMiD-resistant cells, whose sensitivity to lenalidomide or pomalidomide can be restored by selumetinib, a small molecule MEK inhibitor [79].

### Wnt/β-catenin signaling

The conserved Wnt/β-catenin signaling pathway is a key regulator of development, the dysregulation of which is involved in tumorigenesis [80, 81]. Targeting Wnt/β-catenin signaling has been proposed to improve the efficacy of cancer immunotherapy [82]. Dysregulation of Wnt/β-catenin signaling was identified in a lenalidomide-resistant MM cell line [36]. Stimulation of the Wnt/β-catenin pathway can reduce the antimyeloma activity of lenalidomide, while inhibition of β-catenin can restore sensitivity to lenalidomide [36]. This evidence suggests the possibility of targeting Wnt/β-catenin signaling with inhibitors to alleviate IMiD resistance.

### Other factors

Sensitivity to IMiDs has been reported to be affected by factors other than those discussed above. In IMiD-resistant MM cells, dysregulation of a number of signaling mediators has been identified, including upregulation of IL-6/activation of STAT3 [83], increased genome-wide DNA methylation [84], dysregulation of HIF-1α [85, 86], dysregulation of CD44 [87], and decreased CD138 levels [88]. In addition, cellular antioxidative capacity can also affect sensitivity to lenalidomide in MM cells [89]. Activation of c-Abl kinase can potentiate the antimyeloma activity of lenalidomide [90]. RNAi and shRNA screenings have revealed that ribosomal protein S6 kinase (RSK2) and karyopherin beta 1 (KPNB1) are required for lenalidomide and pomalidomide sensitivity in MM cells, respectively [40, 41], while G protein-coupled receptor 68 (GPR68) is essential for lenalidomide sensitivity in del(5q) MDS cells [91].

IMiDs can target the CRL4^CRBN^ E3 ligase to induce the degradation of specific proteins, and each of these compounds has a different spectrum of neosubstrates [29, 47, 48], most of which share a common structural motif containing a key glycine [47, 92–95]. Distinct patterns of substrate specificity may explain the diversity in clinical activity and toxicity of these drugs. For example, degradation of CK1α is a key event for lenalidomide efficacy in del(5q) MDS [46], while GSPT1 degradation is deemed to account for anti-AML activity of CC-885 and CC-90009 [25, 28]. A recent study showed that different neosubstrates compete for CRBN E3 ligase binding in the presence of IMiDs [96]. In this way, IMiD sensitivity is determined by the interplay between the CRBN E3 ligase and a number of potential neosubstrates [96], supporting the key role of CRBN expression in IMiD sensitivity.

## Genome-wide CRISPR screenings as a tool to identify genes required for IMiD sensitivity

### CRISPR genome editing and application

The CRISPR-Cas system, derived from the prokaryotic adaptive immune system, has been modified to be a powerful tool in targeted genome editing [97–99]. CRISPR-Cas9 genome editing is now widely used to generate gene-engineered cell lines and animals in laboratories worldwide [97, 98, 100]. Furthermore, CRISPR-mediated knockout of TCR and HLA class I molecules contributes to the generation of universal CAR-T cells [101–104]. Recent attempts have been made to use the CRISPR-Cas system therapeutically, especially in genetic disorders related to single gene mutations, including sickle cell anemia, cystic fibrosis and Huntington’s chorea [105, 106].

**Fig. 3:**
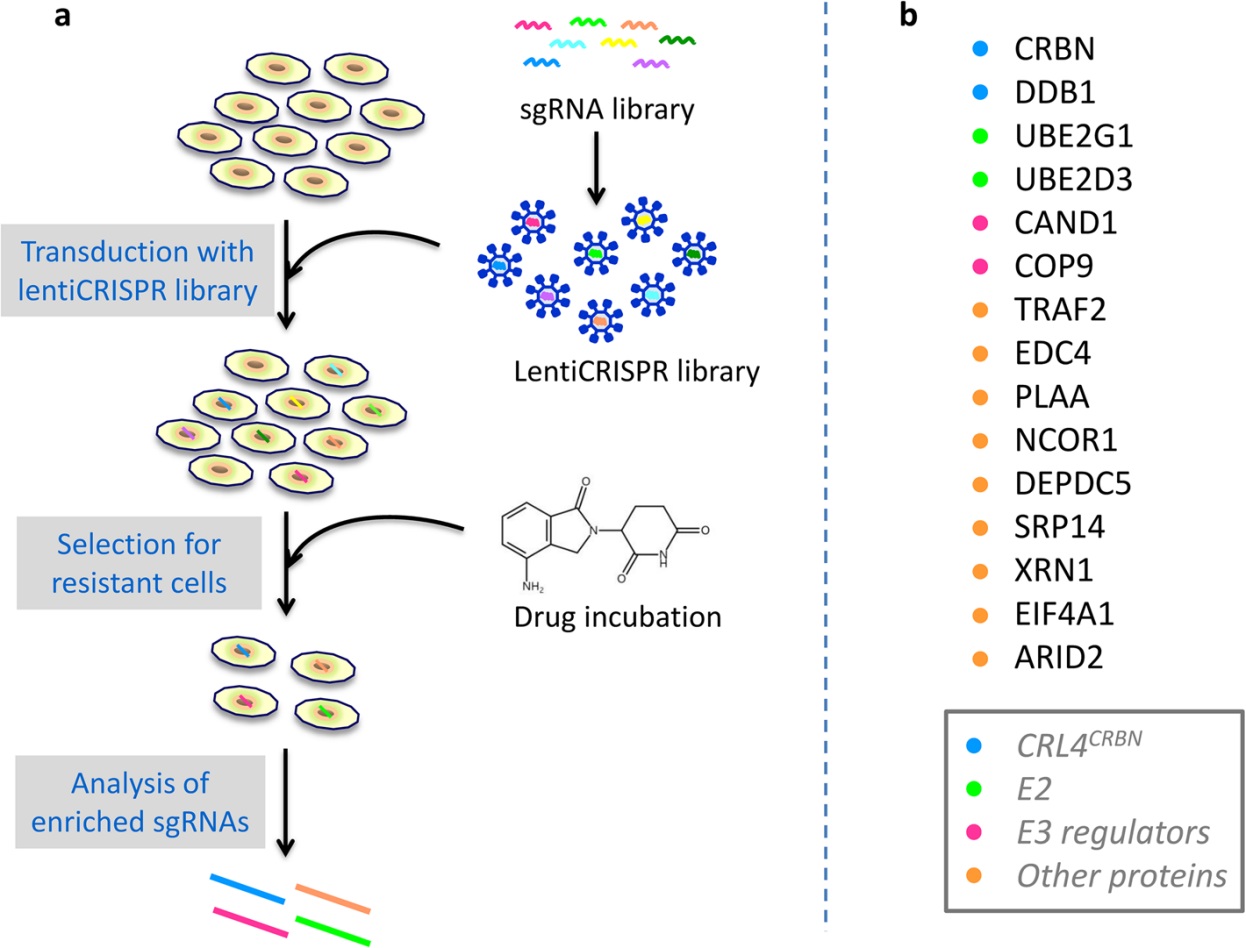
**a** Flowchart of IMiD resistance screenings. A CRISPR sgRNA library targeting different genes is introduced into MM cells via lentiviral vectors, followed by IMiD treatment, and then the cells are collected at different timepoints to analyze enriched sgRNAs. **b** The top ranked genes required for lenalidomide or pomalidomide sensitivity are summarized

In addition to its utility for research on single gene modifications, the CRISPR-Cas system has been applied for large-scale functional screening in genomic, transcriptomic or epigenetic research [107–110]. Genome-wide CRISPR-Cas9 screening has been established to search for critical genes involved in drug resistance (Fig. 3a) [107]. Recently, genome-scale CRISPR screenings have been carried out to identify essential genes required for IMiD sensitivity in MM, primary effusion lymphoma (PEL) and AML [37–39].

### CRISPR-Cas9 screenings to identify genes required for IMiD sensitivity

In one CRISPR-Cas9 screen, a library of sgRNAs targeting 19,050 genes and 1,000 control sgRNAs were introduced into MM1S cells, which were then incubated with lenalidomide or DMSO. Then, these cells were collected and analyzed using next-generation sequencing to identify genes required for lenalidomide activity in MM cells [37]. Among the top 30 genes, 17 were related to CRL4 E3 ligases, including *CRBN*, *DDB1*, subunits of the COP9 signalosome (CSN), *CAND1*, *UBE2G1* and *UBE2D3* [37]. In another CRISPR-Cas9 screen to identify genes required for pomalidomide activity in MM1S cells, a similar subset of targets was discovered [38]. The genes essential for IMiD activity in MM cells are summarized in Fig. 3b.

IMiDs have shown significant efficacy in PEL, a non-Hodgkin B cell lymphoma [111, 112]. A CRISPR-Cas9 screen was conducted in PEL cells to search for genes essential for the activities of lenalidomide, pomalidomide and CC-122 [39]. According to the results, components of the CRL4 machinery were again identified, including *CRBN*, *Cullin 4 A/4B*, *UBE2G1* and *SENP8*, together with other targets [39].

CC-90009, a new CRBN modulator, has shown notable efficacy in AML by selectively inducing degradation of GSPT1. CC-90009 promotes apoptosis of leukemia stem cells in xenografting of 35 primary AML samples, regardless of the adverse risk features [28]. Based on the promising efficacy, CC-90009 has entered clinical trials for AML and MDS (Table 1). A CRISPR-Cas9 screen in U937 cells has revealed essential genes for the efficacy of CC-90009, including subunits of CRL4^CRBN^ E3 ligase, *CSN*, *CAND1*, *ILF2/ILF3* [28].

**Table 1:** Clinical trials of CC-90009

Phase		Conditions	Interventions	NCT ID
1		Healthy Volunteer	CC-90009 Radiation: [14 C]	NCT04297124
1		AML, MDS	CC-90009	NCT02848001
1, 2		AML	CC-90009, Venetoclax, Azacitidine, Gilteritinib	NCT04336982

Abbreviation: *AML* acute myeloid leukemia, *MDS* myelodysplastic syndrome

In summary, components of the CRL4 E3 ligase are required for IMiD sensitivity, which is consistent with the mechanism of these compounds. Furthermore, regulators of CRL4 E3 ligase activity for example, CSN, also affect IMiD activity. Deletion of CSN causes a significant decrease in CRBN protein levels in MM cells, which can explain the IMiD resistance in CSN-deleted cells [38]. AT-rich interactive domain 2 (ARID2), a component of the polybromo-BRG1-associated factors (PBAF) chromatin-remodeling complex, was also identified to be required for pomalidomide activity in MM cells [38], which was recently verified by the discovery of ARID2 as a pomalidomide-induced neosubstrate [113]. Degradation of ARID2 causes downregulation of MYC, leading to the death of MM cells [113]. These data demonstrate the powerful function of CRISPR screens in the discovery of regulatory networks of drug sensitivity.

## Conclusion and perspective

**Fig. 4:**
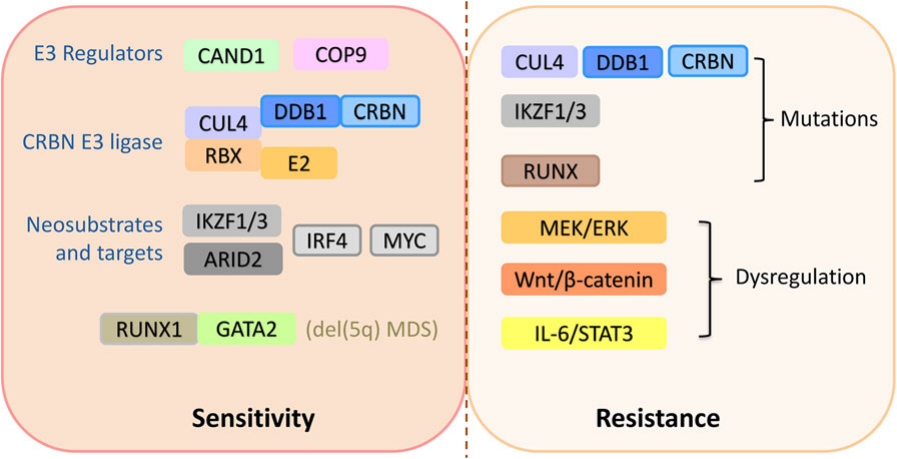
General regulators required for IMiD sensitivity and frequently mutant genes or dysregulated pathways in IMiD-resistant cells. These findings are summarized mainly from studies in MM, or other conditions specified

As the primary target of IMiDs, CRBN is required for IMiD sensitivity. Due to the rapid development of biotechnology tools such as CRISPR genome editing, many other regulators of IMiD sensitivity have been identified. CRL4 components such as Cullin 4 A/4B, DDB1 and E2 and regulators of E3 ligase are also required for IMiD sensitivity. Degradation of IKZF1/3 are essential for antimyeloma activity of IMiDs in MM. RUNX1 and GATA2 are required for lenalidomide activity in del(5q) MDS. Mutations in components of CRL4^CRBN^ E3 ligase, mainly CRBN itself, IKZF1/3 and RUNX have been identified in IMiD-resistant cells, together with dysregulation of MEK/ERK, Wnt/β-catenin and IL-6/STAT3 pathways (Fig. 4). As IMiDs have also shown notable efficacy in different hematological malignancies such as CLL, DLBCL and AML, which have diverse genetic features, disease-specific regulators of IMiD sensitivity might be identified by future studies.

New generation of IMiDs are under clinical development. CC-122 is now in phase 1/2 trials for hematological malignancies, including DLBCL and MM [29]. The most common treatment-emergent adverse events (TEAEs) are neutropenia, thrombocytopenia and anemia [21, 22, 114–116]. CC-220 has shown significant efficacy in systemic lupus erythematosus (SLE) and relapsed/refractory MM (RRMM) and now under phase 1/2 studies [24, 117]. Neutropenia, infection and thrombocytopenia have been reported following CC-220 administration [118–120]. CC-92480 can induce deeper degradation of IKZF1/3, showing therapeutic advantage in lenalidomide-resistant MM cells with little effect on the viability of normal peripheral blood mononuclear cells [27]. CC-92480 is now under phase 1/2 clinical trials mainly for MM. CC-885 has anti-proliferation activity in a broad range of tumor cell lines and significant anti-AML potency by the degradation of GSPT1 [25]. CC-90009 induces the degradation of GSPT1 with higher selectivity and now in phase 1/2 clinical studies for AML and MDS. As these CRBN modulators function through a similar mechanism, the understanding of regulatory networks of IMiD sensitivity may provide reference for the development of new IMiDs.

Proteolysis-targeting chimeras (PROTACs) are bifunctional molecules that can target proteins for degradation via the ubiquitin-proteasome pathway. A typical PROTAC molecule contains a ligand for the protein of interest covalently linked to a moiety of an E3 ubiquitin ligase [121–124]. Since IMiDs repurpose the CRL4^CRBN^ E3 ligase to ubiquitinate and degrade a number of cellular proteins, these molecules have been frequently used in the design of PROTACs. In this way, IMiDs have been linked to ligands of BTK, BCR-Abl, BRD4 and other targets to generate PROTACs that can degrade these oncoproteins [125–127]. Targeting protein for degradation by PROTACs has emerged as a powerful therapeutic strategy in cancer treatment. The discovery of mechanism of IMiDs facilitates the development of PROTACs by providing more choices on E3 ligase utilization. Thus, the delineation of key regulators of IMiD sensitivity may promote the development of IMiD-based PROTACs.

## Data Availability

The material supporting the conclusion of this review has been included within the article.

## Abbreviations

AML: Acute myeloid leukemia
BRD4: Bromodomain-containing protein 4
ARID2: AT-rich interactive domain 2
BTK: Bruton’s tyrosine kinase
CELMoDs: CRBN E3 ligase modulators
CSN: COP9 signalosome
CRL4: Cullin-RING ligase 4
DLBCL: Diffuse large B-cell lymphoma
FL: Follicular lymphoma
GPR68: G protein-coupled receptor 68
IMiDs: Immunomodulatory drugs
KPNB1: Karyopherin beta 1
MCL: Mantle cell lymphoma
MDS: Myelodysplastic syndrome
MM: Multiple myeloma
PEL: Primary effusion lymphoma
PROTACs: Proteolysis-targeting chimeras
RRMM: Relapsed/refractory MM
RSK2: Ribosomal protein S6 kinase
SLE: Systemic lupus erythematosus
TEAEs: Treatment-emergent adverse events
VHL: Von Hippel-Lindau
